# Myocarditis Concurrent with Sweet Syndrome: A Presentation of Acute Myeloid Leukemia

**DOI:** 10.1155/2021/6621007

**Published:** 2021-05-11

**Authors:** Christopher G. Burkeen, David Pottinger, Chaitanya Iragavarapu, Reshma Ramlal, Gerhard Hildebrandt

**Affiliations:** University of Kentucky Markey Cancer Center, 800 Rose Street, Lexington, KY 40536, USA

## Abstract

Acute myeloid leukemia (AML) is the most common acute leukemia in American adults and portends a poor prognosis if untreated. Commonly, AML presents with symptoms related to concurrent leukopenia, anemia, or thrombocytopenia; however, due to its ability to affect many organ systems in the body, AML can have a highly varied clinical presentation. One such presentation is myocarditis, which is a rarely reported manifestation of AML. Myocarditis can have a varied clinical picture and often requires exclusion of other causes of cardiac dysfunction. Sweet syndrome, also known as acute febrile neutrophilic dermatosis, is another presentation of AML; however, it is more commonly associated with AML than cardiac involvement. Sweet syndrome can occur in patients with an already established malignancy or can occur de novo in a patient with previously undiagnosed cancer and, interestingly, can also be accompanied by extracutaneous manifestations, one of which is myocarditis. Herein, we report a case of a 45-year-old male with a history of obesity and depression who presented with chest pain, a tender and diffuse rash, and pancytopenia. Heart catheterization performed at outside institution was negative for coronary artery disease. Cardiac MRI images were compatible with myocarditis. Dermal biopsy of the rash was consistent with sweet syndrome. Peripheral blood flow cytometry and bone marrow biopsy confirmed the diagnosis of AML. He was treated with an induction chemotherapy regimen of 7 days of cytarabine and 3 days of daunorubicin with resolution of his chest pain and skin lesions. The patient had persistent leukemia cells on day 14 postinduction bone marrow biopsy and was treated with high-dose cytarabine reinduction treatment. Bone marrow biopsy with count recovery after reinduction therapy revealed complete response (CR).

## 1. Introduction

Acute myeloid leukemia (AML) is the most common acute leukemia in American adults, accounting for 80% of all leukemia and totaling to a yearly incidence of 20,000 cases in the US [1]. AML is known to have a highly varied clinical presentation due to its ability to infiltrate and affect many organ systems in the body. One such association is a clinical entity known as sweet syndrome, which is also known as acute febrile neutrophilic dermatosis. Sweet syndrome is characterized by discrete, tender skin lesions with a heavy infiltrate of mature neutrophils and may be accompanied by extracutaneous disease. In up to 20% of cases, sweet syndrome is associated with malignancy with AML being the most frequent [2]. It can occur in patients with established diagnosis of malignancy as a paraneoplastic disorder, or it could occur prior to a formal diagnosis of malignancy. Furthermore, one potential extracutaneous manifestation of sweet syndrome is myocarditis, which is also a rare presentation of AML. Autopsies of patients who expired due to AML have shown that up to 37.1% have cardiac leukemic infiltrates [3]. However, myocarditis is a very uncommon manifestation of this, with our literature review producing only three case reports describing this association with AML. Our literature review also produced only four cases of patients with concurrent sweet syndrome and myocarditis. Here, we present a patient who presented with chest pain and increased cardiac enzymes secondary to myocarditis, a rash that was biopsy proven to be sweet syndrome, and pancytopenia secondary to new diagnosis of AML.

## 2. Case Presentation

A 45-year-old male with a past medical history of hypertension and depression presented to an outside hospital's emergency department with a primary complaint of chest pain of one day duration. He was in his usual state of health prior to the onset of the chest pain. This chest pain was progressively worsening, substernal, radiated to his back, and was unaffected by position. On presentation, he was found to have elevated troponins and was provided nitroglycerin, which relieved his chest pain. Initial electrocardiogram (ECG) was without any ST elevations. He was subsequently taken for left heart catheterization and found to have nonobstructive coronary artery disease. He was discharged to home on metoprolol, aspirin, statin, and a proton pump inhibitor. He was also discharged on indomethacin due to suspicion of myocarditis at that time. After discharge, however, his chest pain continued to worsen. Around that time, he also started to notice a painful dark rash on his legs.

The worsening chest pain and new onset rash prompted him to return to the ED about five days after his recent discharge, and he was subsequently transferred to our hospital's ED. On presentation, he further endorsed strong feelings of fatigue over the prior few days and was admitted to several nights of drenching sweats. He also had subjective fevers and chills. He denied any recent shortness of breath, documented fevers, changes in weight, or sensation of heart palpitations. The patient was afebrile with a heart rate of 76, blood pressure of 129/63, a respiratory rate of 18, and 99% oxygen saturation in room air. On physical exam, the patient was an obese male in no acute distress. He was observed to have a linear grouping of well-circumscribed tender papules with a deep red to purple base on his left thigh with the largest lesion being about six centimeters. He also had similar smaller lesions located on his right forearm, left upper arm, and left cheek. Cardiopulmonary exam was normal with clear lungs in all fields and no abnormal heart sounds or peripheral edema. The rest of systemic examination was normal.

**Figure 1:**
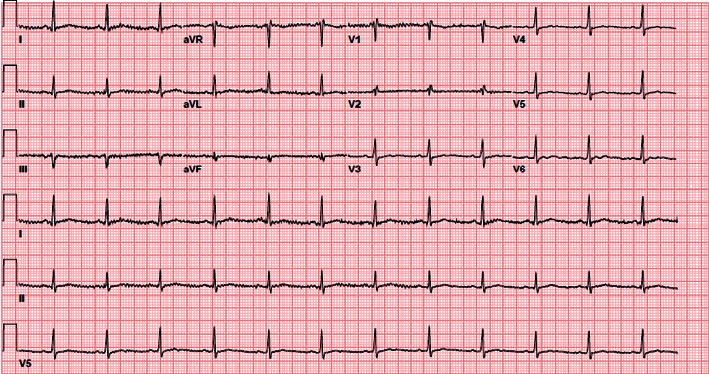
EKG performed in the ED showed a heart rate of 74 and a regular sinus rhythm, with no acute disease processes.

Initial laboratory evaluation revealed elevated high-sensitivity troponins at 958 ng/L (<19 ng/L) and elevated NT-proBNP at 1104 pg/mL (<449 pg/mL). Repeat high-sensitivity troponin level 2 hours later was stable at 899 ng/L. His initial ECG showed no acute changes (Figure 1), and a bedside echocardiogram showed a preserved ejection fraction.

**Figure 2:**
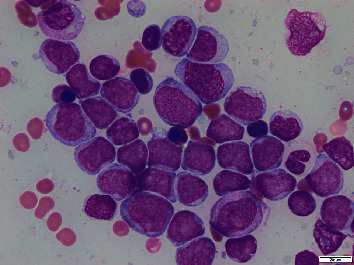
Bone marrow aspirate smears were significant for approximately 62% myeloblasts, and the background hematopoiesis was markedly decreased.

Continued workup revealed a white blood cell count of 2.20 k/*µ*L (3.7–10.3 k/uL), an absolute neutrophil population of 0.75 k/*µ*L, 21% peripheral blasts, hemoglobin of 8.7 g/dL (13.7–17.5 g/dL), and a platelet count of 116 k/*µ*L (155–369 k/uL). This pancytopenia with peripheral blasts and an elevated LDH of 335 U/L (116–250 U/L) raised strong suspicion for acute leukemia. His uric acid, creatinine, and liver function tests were all within normal limits. Peripheral blood smear confirmed pancytopenia with circulating blasts. Peripheral blood flow cytometry was significant for expanded myeloid blast population with 19% of the cells. Subsequent bone marrow biopsy confirmed acute myeloid leukemia with 62% blasts by flow cytometry in a hypercellular marrow with markedly decreased background hematopoiesis. Immunophenotype showed blasts expressing CD34, CD117, partial CD13, dim CD33, HLA-DR, CD7, CD38, and moderate to dim CD45. Bone marrow aspirate smear is shown in Figure 2. Molecular testing for NPM1 and FLT3 was negative. FISH panel was positive for t(11q23) and gain of 3′KMT2A, diagnosing him with poor-risk mixed leukemia lineage- (MLL-) associated AML.

**Figure 3:**
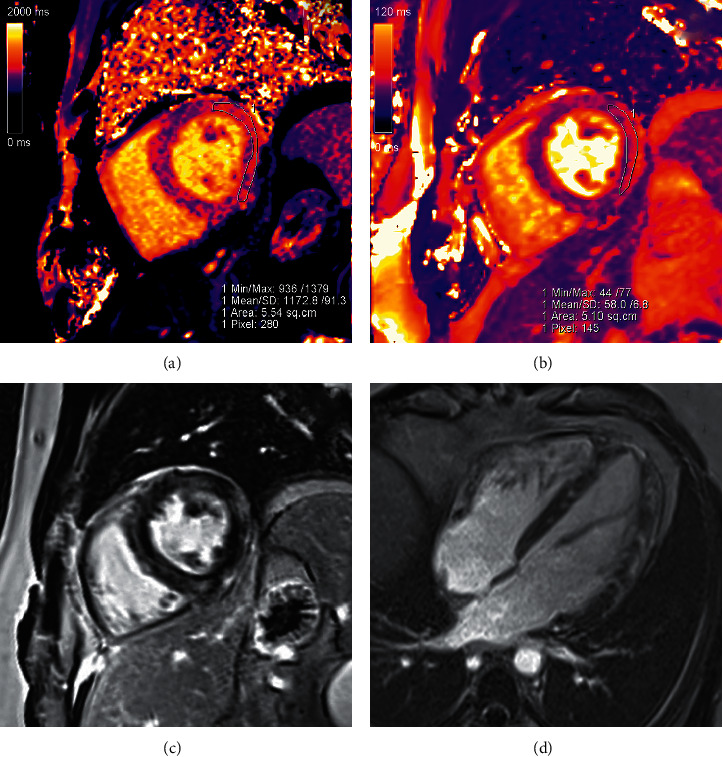
Cardiac MRI showed features consistent with myocarditis satisfying the updated Lake Louise criteria [4], visualized in these images. (a) Mid-short-axis native T1 mapping demonstrated elevated native myocardial T1 at 1173 ms (local lab normal reference: 950–1050 ms). (b) Mid-short-axis T2 mapping demonstrated elevated myocardial T2 at 58 ms (normal reference: 40–50 ms). (c) Mid-short-axis phase-sensitive inversion recovery late gadolinium enhancement image showed multiple areas of subepicardial enhancement in the lateral wall, as well as midmyocardial enhancement in the septum. (d) Four-chamber late gadolinium enhancement image demonstrated midmyocardial enhancement in the septum and subepicardial to midmyocardial enhancement in the lateral wall.

**Figure 4:**
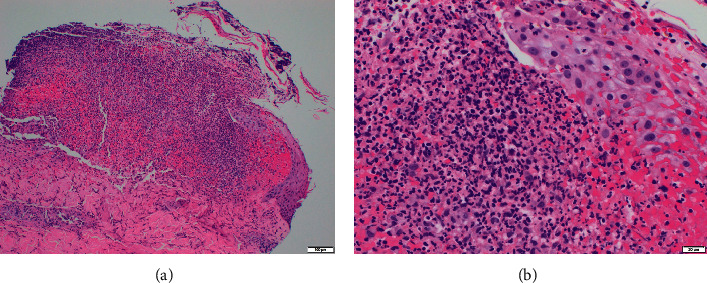
Skin biopsy showed dense dermal infiltrate of polymorphonuclear cells at both low (a) and high (b) magnification.

A dedicated cardiac MRI (Figure 3) demonstrated evidence of nonischaemic myocardial inflammation consistent with myocarditis. It also demonstrated multiple areas of late subepicardial gadolinium enhancement in the lateral wall and midmyocardial enhancement in the septum. Of note, the MRI showed a normal ejection fraction and no associated pericardial effusion. CMV PCR, coxsackie viral PCR, adenovirus PCR, Lyme serology, anaplasmosis serology, ehrlichiosis serology, mycoplasma PCR, influenza A and B testing, COVID-19 PCR, HIV screen, and hepatitis testing were negative. Furthermore, syphilis RPR, ANA, ANCA, and blood and urine cultures were also negative. With these negative tests, a presumptive diagnosis of AML-related myocarditis was made via exclusion. A biopsy of the patient's skin lesions was then performed, revealing for a “dense dermal infiltrate of polymorphonuclear cells in a pattern that would fit best with a neutrophilic dermatosis,” qualifying a final diagnosis of sweet syndrome. The histologic image of the skin biopsy at both low and high magnification is shown in Figure 4.

The patient was started on “7 + 3” induction chemotherapy with seven days of cytarabine dosed at 100 mg/m^2^ daily and three days of daunorubicin dosed at 60 mg/m^2^ daily. The patient had complete resolution of his chest pain shortly thereafter and marked improvement in his skin lesions. His day 14 postinduction bone marrow biopsy showed 60% cellularity with 50% blasts consistent with prior leukemic blasts by flow cytometry. Due to persistent presence of leukemia, he was started on high-dose cytarabine (HiDAC) reinduction chemotherapy. After HIDAC, the patient had count recovery, and bone marrow biopsy at that time revealed no evidence of acute myeloid leukemia; therefore, he was determined to have complete response. He was discharged from the hospital seven weeks after his admission date. At the time of discharge, his skin lesions had healed to a significant extent.

## 3. Discussion

Acute myeloid leukemia has a highly variable clinical presentation with rare associations documented. AML-related myocarditis is one such poorly studied disease entity. In general, myocarditis is a disease secondary to inflammation of the cardiac muscle and has a nonspecific clinical presentation that ranges from acute chest pain to new onset heart failure to cardiogenic shock, among others. Diagnosis of clinically suspected myocarditis is based on a combination of the clinical presentation and diagnostic findings, which can include characteristic tissue imaging on cardiac MRI [5]. This diagnosis via cardiac MRI is established via the updated Lake Louise criteria where both of the following criteria must be met: demonstration of edema, with regional or global myocardial signal intensity increase in T2-weighted images or with prolonged T2 relaxation times on native T2 mapping, as well as late gadolinium enhancement in a nonischaemic distribution on inversion recovery-prepared gadolinium-enhanced T1-weighted images [4]. Often, coronary arterial disease has to be ruled out via angiography. Of course, gold standard diagnosis of myocarditis is based on endomyocardial biopsy [6]. Infiltration of the heart muscle by malignant leukemia cells is a known sequela of AML, but this process is usually subclinical for unknown reasons [3]. Of 420 autopsies of myelogenous or lymphocytic leukemia patients evaluated by Roberts et al., 288 (69%) of patients had some degree of leukemic involvement of the heart. 190 of these patients had pericardial involvement, which was the most common cardiac site of involvement [3]. AML with an initial presentation of pericarditis is estimated to occur in 1-2% of cases. Myocarditis, however, is less frequently documented. In our literature review, we identified only three previous case reports documenting AML-related myocarditis, which are described in Table 1 [7–9]. Of note, pericarditis was also present in two of the three cases. Furthermore, two of the three patients had cardiac MRI with characteristic findings, and all three patients had negative coronary artery angiography.

In contrast, acute myeloid leukemia is associated with dermatologic involvement more commonly, with presence in about 10% of new cases. These skin lesions include erythematous nodules, papules, or plaques, among others [10, 11]. One of these dermatologic associations is an entity known as sweet syndrome. The classical form of sweet syndrome, also known as neutrophilic dermatosis, is a rare disorder consisting of fever, neutrophilia, and tender erythematous skin lesions characterized by mature neutrophils in the upper dermis [12]. Numerous precipitating factors have been identified, including inflammatory bowel disease, pregnancy, and administration of drugs such as G-CSF, among others. Sweet syndrome cases are very often associated with malignancy, with AML being the most common offender. Malignancy-associated sweet syndrome has been documented on numerous occasions to be the presenting symptom of previously undiagnosed neoplastic process; however, it can also occur as a paraneoplastic process in a patient with an already established diagnosis of malignancy. The exact pathogenesis of sweet syndrome is not fully understood; various proposed mechanisms have included an exaggerated hypersensitivity reaction, heritable mutations predisposing to the illness, and dysregulated cytokine (especially G-CSF) production. The lesions classically appear in multiple locations on the body, with one study suggesting that the upper and lower extremities and trunk were most commonly affected [13]. This study also found that a majority of the lesions were erythematous, with plaques being the most common morphology, but with vesicles or pustules being a known variant of the disease, appearing just under 20% of the time. The lesions were tender to palpation in 43% of cases and pruritic in 18% of cases. The gold standard for diagnosis is a biopsy showing the characteristic diffuse infiltration by mature neutrophils. First-line treatment is systemic corticosteroids, which typically cause immediate improvement in symptoms, with subsequent resolution of skin lesions [13]. Treatment of the underlying cause, if identified, is also indicated.

Our patient's presentation was consistent with malignancy-associated sweet syndrome in several ways. The papular lesions were tender, erythematous, and characteristically distributed on his lower and upper extremities. His previously undiagnosed AML is the most common malignancy associated with sweet syndrome. A skin biopsy ultimately confirmed the disease, and his rash improved with treatment of his malignancy. This patient's concurrent myocarditis diagnosis was established with cardiac MRI using the updated Lake Louise criteria [4]. His myocarditis was likely related to his diagnosis of sweet syndrome due to exclusion of other etiologies and occurring in the setting of both sweet syndrome rash and acute leukemia, two known associations. The treating physicians still decided to treat with an anthracycline-based induction chemotherapy regimen despite the finding of myocarditis. This was because the patient still had preserved heart function, was hemodynamically stable, and was of young age with few comorbidities and thus would most benefit from high-intensity induction chemotherapy. Fortunately, his chest pain never recurred after treatment of his leukemia. Cases in the literature regarding the combination of sweet syndrome and myocarditis are described in Table 2 [14–17]. All four patients were treated for their sweet syndrome with some form of steroid administration. Three of the four had complete recovery from their disease; however, the fourth died due to cardiac arrest shortly after presentation. Of significance, there are no reported cases of sweet syndrome with concurrent myocarditis as symptoms of acute myeloid leukemia.

In conclusion, this patient had biopsy-proven neutrophilic dermatosis and subacute myocarditis as presenting symptoms of acute myeloid leukemia, supporting a diagnosis of sweet syndrome. Providers should be aware of this clinical syndrome, especially because it may be the first manifestation of underlying hematologic malignancy.

## Figures and Tables

**Table 1:** Myocarditis/myopericarditis in AML patients.

Age/sex	Presentation	Cardiac evaluation	Leukemic evaluation	Treatment	Author
37/F	Acute exertional chest pain with radiation to arms.	Negative coronary artery angiography. TTE: EF of 50–55% with hypokinetic right ventricle. Cardiac MRI: EF of 33% with signal changes consistent with myocarditis.	AML “confirmed on bone marrow biopsy.”	Myopericarditis: colchicine and NSAIDs.	Snavely et al. [7]
	ECG with ST elevations in leads I, aVL, V1, and V2 and PR elevation in lead AVR. Troponin elevated at 1.6 ng/mL.			AML treatment not stated.	
	CBC showed WBC of 13.7 k/*µ*L with 28% blasts, Hgb of 10.0 g/dL, and Plts 145 k/*µ*L.				

48/M	Acute substernal chest pain. Febrile (103.4°F).	Negative coronary artery angiography with an ejection fraction of 40–45%. There was mild hypokinesis in the midinferior and mid-anterolateral walls and no pericardial effusions. TTE revealed the same findings.	Bone marrow biopsy: hypercellular marrow with diffuse mononuclear infiltrates with flow cytometry consistent with AML. FISH analysis was positive for t(6; 9) (p23; q34).	Myopericarditis: colchicine 0.6 mg BID; ibuprofen 600 mg TID	Agrawal et al. [8]
	ECG with ST segment elevation in leads I, II, aVL, and V_5_ with PR elevation and ST depression in aVR. Troponin-I was 14.8 ng/mL. CBC revealed hemoglobin of 10.8 g/dL, white cell count of 13,000/*µ*L, 56% blasts, absolute neutrophil count of 1,430/*µ*L, and platelet count of 83 × 10^3^/*µ*L.			AML: 7 d cytarabine and 3 d idarubicin followed by salvage therapy with HIDAC and midostaurin.	

62/M	Acute chest pain. ECG with ST elevation in inferior leads. Troponin of 1.5 *µ*g/L.	TTE: normal biventricular function with inferolateral hypokinesia. Angiography: normal coronary arteries.	Bone marrow biopsy: 47% blasts suggestive for AML.	Myocarditis: anti-inflammatory therapy.	De Lazzari et al. [9]
		Cardiac MRI: cardiac dysfunction with areas of myocardial edema and late enhancement.		AML: fludarabine, steroids, and cytarabine.	

AML: acute myeloid leukemia; CBC: complete blood count; ECG: electrocardiogram; EF: ejection fraction; FISH: fluorescence in situ hybridization; Hgb: hemoglobin; HIDAC: high-dose cytarabine; Plts: platelets; TTE: transthoracic echocardiogram; WBC: white blood cells.

**Table 2:** Cases of sweet syndrome with concurrent myocarditis.

Age/sex	Suspected etiology	Factors supporting diagnosis of sweet syndrome	Factors supporting diagnosis of myocarditis	Therapy administered	Outcome	Author
40/F	Mesalamine (drug induced)	Edematous and erythematous plaques with some central blistering and erosions, located on the arm, neck, and palate.	Fever, acute shortness of breath, and chest pain.	Topical clobetasol cream, 6 weeks of indomethacin + colchicine, and mesalamine discontinued	Resolution of myopericarditis within 20 days; no skin lesions at 6 weeks of follow-up	Shabtaie et al. [14]
		Biopsy: inflammatory infiltrate of the dermis, consisting of neutrophils and leukocytoclasis.	Troponin T 0.12 ng/mL (elevated) and CRP 184 mg/L (elevated).			
			ECG: diffuse ST elevations.			
			Cardiac MRI: diffuse pericardial enhancement and edema.			

64/F	Myelodysplastic syndrome	Movable, tender, erythematous, cutaneous nodule of 1.5 cm × 1.5 cm at the inner aspect of the right lower leg. Skin biopsy demonstrated acute febrile neutrophilic dermatosis.	Clinically, the patient had symptoms consistent with myocarditis.	Prednisolone 30 mg/day	Death secondary to sudden cardiac arrest 3 days after presentation	Shimizu [15]
			Cardiac MRI: a small amount of pericardial effusion and slightly dilated LA. Postmortem heart biopsy: perivascular and myocardial neutrophil infiltration.			

41/M	Idiopathic	Painful erythematous papules and plaques on the nape, neck, shoulders, and arms, as well as painful hyperpigmented subcutaneous nodules. Skin biopsy: subepithelial edema, dermal inflammatory infiltrate with polymorphonuclear predominance and absence of vasculitis.	EKG changes and elevated cardiac biomarkers.	Oral prednisolone 1 mg/kg/day	Complete resolution of symptoms within 4 days of presentation	Graça-Santos et al. [16]
			Cardiac MRI: patchy subepicardial enhancement and slight edema in the inferolateral wall.			

42/M	G-CSF administration	“New skin lesions compatible with sweet syndrome reactivation.” Sweet syndrome had previously been confirmed on biopsy in an episode 1 year prior.	Myocardial biopsy revealed interstitial edema and perivascular neutrophil infiltrate. Additionally, an echocardiogram with new LV systolic dysfunction and CXR with enlarged cardiac silhouette.	Hydrocortisone 1 mg/kg/day, with noninvasive ventilation and IV diuretics	Clinical parameters, invasive monitoring values, and ventricular function normalized at 48 hours	Díaz et al. [17]

CXR: chest X-ray; CRP: C-reactive protein; ECG: electrocardiogram; LA: left atrium; LV: left ventricle.

## Data Availability

The literature review data used to support the findings of this study are included within the article.
